# Ovarian Steroids Mediate Sex Differences in Alcohol Reward After Brain Injury in Mice

**DOI:** 10.3389/fnbeh.2022.907552

**Published:** 2022-06-21

**Authors:** Robin Oliverio, Julie Fitzgerald, Ruth Velazquez-Cruz, Bailey Whitehead, Kate Karelina, Zachary M. Weil

**Affiliations:** ^1^Department of Neuroscience and Rockefeller Neuroscience Institute, West Virginia University, Morgantown, WV, United States; ^2^Department of Neuroscience, Ohio State University, Columbus, OH, United States

**Keywords:** traumatic brain injury, alcohol, sex steroids, organizational effects, androgens, ovaries

## Abstract

Intoxication is a leading risk factor for injury, and TBI increases the risk for later alcohol misuse, especially when the injury is sustained in childhood. Previously, we modeled this pattern in mice, wherein females injured at postnatal day 21 drank significantly more than uninjured females, while we did not see this effect in males. However, the biological underpinnings of this sex difference have remained elusive. In this study, we utilize this preclinical model and traditional endocrine manipulations to assess the effect of perinatal sex steroids on post-injury ethanol response. We found that perinatal androgen administration and adult ovariectomy prevented the development of conditioned place preference to ethanol in females, while there was not an effect of gonadectomy either developmental time point on the severity of axonal degeneration. Finally, although TBI increased the number of microglia in males, there was no corresponding effect of gonadectomy, which suggests that males exhibit prolonged neuroinflammation after brain injury irrespective of circulating sex steroids. Taken together, our results indicate a potential role for ovarian sex steroids in the development of greater alcohol preference after a juvenile TBI in female mice.

## Introduction

The rate of traumatic brain injury (TBI) is notably increasing, with the latest report suggesting that 2.8 million individuals experience a TBI in the United States annually compared to 1.7 million according in a 2011 study (Coronado et al., 2011; Taylor et al., 2017). Intoxication is a leading risk factor for traumatic brain injury (TBI), and alcohol use disorder (AUD) is the most commonly diagnosed psychiatric disorder among TBI patients (Whelan-Goodinson et al., 2009). Moreover, the relationship between TBI and alcohol is bidirectional. Recent studies have suggested that TBI itself might increase the risk of future alcohol misuse (Adams et al., 2012; Corrigan et al., 2014; Wu et al., 2016). By some estimates more than half of substance abuse patients seeking treatment have a history of TBI (Sacks et al., 2009). Therefore, drinking increases the risk of TBI and TBI increases drinking, leading to a destructive cycle. Importantly, little is known about how injury itself increases the risk of developing AUD.

This phenomenon is most prominent in those who experienced a TBI in childhood (Corrigan et al., 2013). For many reasons, it is vital to study this population, as children comprise a large subset of those effected by TBI, and TBI is a leading cause of death and disability among this age group (Langlois et al., 2006; Coronado et al., 2011). Additionally, the TBI population is more likely to experience anxiety, unemployment, and substance use disorders in general (Adams et al., 2012; Karver et al., 2012; Ilie et al., 2015).

Our lab previously reported sex differences in alcohol-related behaviors in adult mice that experienced a mild TBI early in life. Female mice injured at 21 days-old drank significantly more than uninjured females; however, this effect was not observed in males (Weil et al., 2016b). Moreover, injured female mice developed a conditioned place preference to ethanol, whereas males and uninjured females did not. Importantly, these findings resemble epidemiological data which indicate women who experience a TBI in adolescence are much more likely to misuse alcohol than women injured at any age, while this age-related distinction is not observed for men (Corrigan et al., 2020). Although there are sex differences noted in the epidemiological and preclinical literature surrounding TBI, most studies of TBI have focused exclusively on males, and this has left the consequences of injury among women largely understudied. This neglect is especially problematic as there are some indications that women fare worse after injury, including needing surgical intervention more often than men and exhibiting greater durations of posttraumatic amnesia and hospitalization (Farace and Alves, 2000; Oliverio et al., 2020). Given the mounting evidence that TBI can also increase the risk of AUD, this further highlights the need to address this gap in the literature. Of note, although women are less likely than men to receive a diagnosis of AUD, this disparity appears to be decreasing (Keyes et al., 2008), and women face numerous risks as they tend to progress through the stages of addiction at a faster rate, experience greater impact to their health, and are less likely to receive proper treatment (Foster et al., 2014; Mccrady et al., 2020).

The biological mediators of this sex difference have remained unknown. Noting that these studies have revealed sex differences in alcohol-related behaviors, many of which do not emerge until puberty, we postulate that sex hormones are involved in this process. Within the neuroendocrine literature, sex steroid functions are categorized into organizational and activational roles. Organizational effects generally occur early in development and establish the capacity of the nervous system to promote male-typic or female-typic reproductive behaviors later in life. Perinatal production of androgens by the testes (and subsequent aromatization to estrogens within the nervous system) results in the masculinization and de-feminization of both the external genitalia and the nervous system. In contrast, it is the absence of androgens in females which feminizes and de-masculinizes the CNS. Activational effects occur later in life when sex steroids modulate sex-typical reproductive behaviors. Alterations, in the organizational aspect of sex steroids affects the activational component of sex steroids. For instance, the introduction of androgens perinatally in females will prevent ovarian cyclicity (Gorski, 1973). Here, we hypothesized that sex differences in alcohol seeking behavior after TBI are driven in part by organizational and/or activational effects of sex steroids. To test this, we manipulated sex steroids through perinatal androgen exposure in females and gonadectomies in males and performed a mild closed-head injury early in life. In adulthood, we assessed activational effects of sex steroids on alcohol-related behavior via gonadectomy. We report that while injury severity was not altered by these sex steroid manipulations, perinatal androgen exposure and adult gonadectomy in females prevented the development of a conditioned place preference following injury. Taken together, our data indicate that sex steroids play a role in moderating alcohol-related behavior following a juvenile TBI.

## Materials and Methods

### Animals

Swiss-Webster mice were purchased from Charles River (Wilmington, MA) and bred at Ohio State University (OSU). Pups were weaned at postnatal day (PND) 21 and housed in standard mouse cages under a 14:10 light-dark cycle. Mice were provided with access to food and water *ad libitum*, and all experiments were performed in accordance with approval from the OSU Institutional Animal Care and Use Committee.

### Hormone Manipulations

To measure organizational effects of sex steroids, neonatal pups underwent hormone manipulations. Female mice were injected subcutaneously with testosterone, 100mg (per pup) crystalline testosterone (Sigma Aldrich) dissolved in 100 μL olive oil (Thermo Scientific) or oil alone on PND 4 (Klein et al., 2002). Male mice were gonadectomized (GDX) under cryoanesthesia at PND 4 or underwent a sham operation (Anderson et al., 2005). Briefly, the skin was disinfected with alternating swabs of betadine and 70% ethanol, a vertical midline incision was made, and the testes were removed with forceps. The incision was sutured, and pups were returned to their home cage on a heating pad. The sham procedure included an incision but no manipulation of the gonads. To measure activational effects of sex steroids, mice underwent GDX or control procedures at PND 60 as previously reported (Aubrecht et al., 2015) producing the following groups: females (oil + control “control”, oil + GDX “GDX”, T + GDX), males (control, neonatal GDX, adult GDX). Note that to control for age differences at the time of surgery, all male mice underwent two surgical procedures (at PND 4 and PND 60), undergoing either two control surgeries, or one control and one GDX. All mice were randomly assigned to each group.

### Traumatic Brain Injury

**FIGURE 1:**
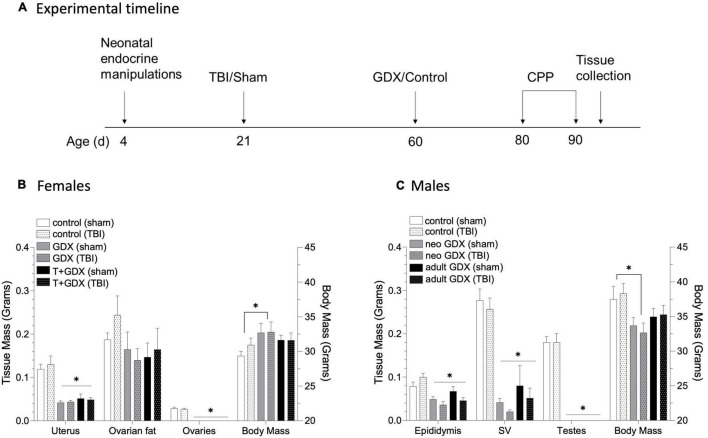
Timeline of experimental procedures and tissue responses to endocrine manipulation. **(A)** Male and female mice underwent neonatal endocrine manipulations at PND 4 (T or oil for females, GDX or control surgery for males). TBI or sham surgery was conducted at PND 21. Additional endocrine manipulations were conducted at PND 60 (females underwent GDX or control surgery, males that underwent early life control surgery had either GDX or control surgery in adulthood, and males that underwent early life GDX had a control surgery). Females (control/sham *n* = 9, control/TBI *n* = 9, GDX/sham *n* = 8, GDX/TBI *n* = 8, T + GDX/sham *n* = 9, T + GDX/TBI *n* = 10), Males (control/sham *n* = 7, control/TBI *n* = 7, neonatal GDX/sham *n* = 7, neonatal GDX/TBI *n* = 7, adult GDX/sham *n* = 8, adult GDX/TBI *n* = 8). All mice were tested for conditioned place preference beginning PND 80. **(B)** Uterine and ovarian tissue masses and body mass in female mice and **(C)** epididymal, seminal vesicle and testicular tissue masses and body mass in male mice. An asterisk (*) indicates statistically significant difference from the relevant control group within each sex, ANOVA *p* < 0.05. Data are presented as mean ± SEM.

A mild closed head injury (or sham procedure) was performed on mice at PND 21. Mice were anesthetized with isoflurane at 3% for induction and 1.5% during the operation. The injury was performed using an Impact One device (Leica Biosystems, Richmond, IL) while the head was stabilized in the stereotaxic frame. Bupivacaine (1mg/kg) was injected SC at the incision site, the skull was exposed, and the device impactor was placed on its surface at –1 AP and 1 ML relative to Bregma. The 2mm diameter tip of the impactor was then depressed 1mm into the skull at 3m/sec with a 30msec dwell time. The process was the same for sham mice, excluding the depression of the plunger. Mice were then returned to their homecage, and additional endocrine manipulations were performed at PND 60 as discussed above. The following groups were generated: Females (control/sham *n* = 9, control/TBI *n* = 9, GDX/sham *n* = 8, GDX/TBI *n* = 8, T + GDX/sham *n* = 9, T + GDX/TBI *n* = 10), Males (control/sham *n* = 7, control/TBI *n* = 7, neonatal GDX/sham *n* = 7, neonatal GDX/TBI *n* = 7, adult GDX/sham *n* = 8, adult GDX/TBI *n* = 8). See Figure 1A for the timeline of hormonal manipulations, TBI (or sham surgery), and behavioral analyses.

### Conditioned Place Preference

The ethanol CPP protocol (adapted from O’Neill et al., 2013; Cunningham, 2014) beginning PND 80 as follows. A plastic container held two chambers of different tactile (thick and thin grid) and visual patterns (checkered and lined) that were separated by a barrier with a small passageway. On the first day, mice were injected intraperitoneally with saline (10mL/kg) and allowed to cross freely between the chambers for 5 min to allow the mice to habituate and determine baseline side bias. Treatment/cue pairings were counterbalanced and randomly assigned. For days 2, 4, 6, and 8, mice were injected with a 20% ethanol solution (2 g/kg), returned to their home cages for 5 min, then confined to an assigned side of the container for 5 min. On alternating days (3, 5, 7, and 9), mice were injected with saline and confined to the opposite-patterned chamber for 5 min. Finally, on the 10th day, mice were injected with saline and allowed to travel freely between the chambers. Conditioned place preference was determined by assessing the amount of time spent exploring the chamber associated with ethanol on day 10 relative to day 1.

### Tissue Processing

Tissue was collected after transcardial perfusion with 4% paraformaldehyde after mice were overdosed with sodium pentobarbital. The forebrain was sectioned coronally into 40 μm slices. Immunohistochemistry was performed for the microglia-specific protein Iba1 using an antibody purchased from Wako (1:1000 anti-Iba1, rabbit) as previously reported (Karelina et al., 2017). Briefly, tissue was washed with 0.1M phosphate buffered saline, quenched with hydrogen peroxide, and incubated overnight with anti-Iba1 antibody. The next day tissue was incubated with a goat anti-rabbit biotinylated secondary antibody (1:500) and visualized using the ABC-DAB method. To assess axonal degeneration, a silver stain was performed with the FD NeuroSilver™ Kit II from FD Neurotechnologies following the manufacturer’s instructions (Columbia, MD).

### Microscopy

Photomicrographs of Iba1 staining were obtained at a 20X magnification (Nikon E800 microscope) and cell counts were conducted using FIJI (Schindelin et al., 2012). Cell counts were obtained from defined regions of interest (ROI) (prefrontal cortex, ROI = 0.04 mm^2^; amygdala, ROI = 0.04 mm^2^; nucleus accumbens, ROI = 0.072 mm^2^), averaged across the hemispheres, and reported as cells per mm^2^. Axonal degeneration was observed based on silver staining in the white matter tracts of the forebrain. The silver score was determined as previously reported qualitatively using a point-value system: such that 0 = little to no axonal degeneration, 1 = sparse silver staining limited to the corpus callosum, 2 = moderate silver staining in the corpus callosum and other white matter tracts, and 3 = very dense silver staining throughout multiple white matter tracts (Karelina et al., 2021). All analyses were performed blind to experimental conditions.

### Statistical Analysis

Statistical analysis was performed using SPSS Version 26 (IBM Corp., Armonk, NY, United States). CPP responses, silver staining, and microglial immunohistochemistry were assessed separately for males and females via a two-way ANOVA (injury x endocrine manipulation). Reproductive tissue and body masses were assessed via a two-way ANOVA (injury x endocrine manipulation). Significant overall ANOVA results were followed up by a one-way ANOVA (factor = injury) between specified groups for CPP, or a Tukey HSD *post hoc* test for reproductive tissue and body mass. Effect sizes are reported as partial eta squared (η_p_^2^). Results are considered significant when *p* ≤ 0.05.

## Results

### Perinatal Testosterone Administration and Gonadectomies Reduce the Masses of Steroid-Sensitive Tissue

To determine the role of circulating sex steroids on alcohol-related behavior after brain injury, we manipulated gonadal steroid availability at critical developmental periods (perinatally and at adolescence). The effectiveness of our endocrine manipulations was verified by assessing reproductive tissue masses at necropsy (see Figure 1 for time line). Endocrine manipulations altered tissue masses, but injury did not (*p* > 0.5 for all sham vs. TBI comparisons). Uterine mass was reduced in GDX and T + GDX females compared to control mice (F_2,51_ = 34.74, *P* < 0.00001, η_p_^2^ = 0.591), ovarian fat pad mass was also reduced but not significantly (F_2.50_ = 20.154, *P* = 0.14, η_p_^2^ = 0.127). Body mass was also altered by endocrine manipulations (F_2,52_ = 3.141, *P* = 0.05, η_p_^2^ = 0.114) such that GDX females were heavier than controls. For males, epididymides (F_2, 43_ = 13.874, *P* < 0.00001, η_p_^2^ = 0.41) and seminal vesicle masses (F_2,42_ = 48.985, *p* < 0.00001, η_p_^2^ = 0.715) were reduced in NEO GDX and Adult GDX groups. Body mass was altered by endocrine manipulations (F_2,41_ = 6.064, *p* = 0.005, η_p_^2^ = 0.228) such that NEO GDX male mice were lighter than intact mice.

### Sex Steroid Manipulation in Females Prevents Development of Conditioned Place Preference Following Injury

**FIGURE 2:**
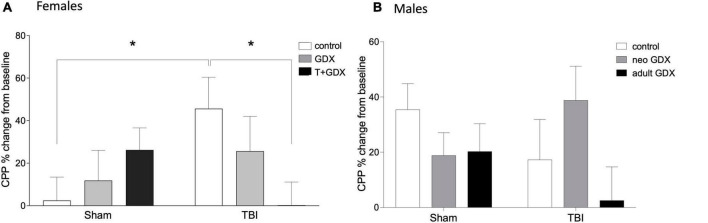
Conditioned place preference to ethanol. Percent change of conditioned place preference to alcohol from baseline in **(A)** female and **(B)** male mice. An asterisk (*) indicates statistically significant difference from control/TBI females, ANOVA *p* < 0.05. Data are presented as mean ± SEM.

An overall ANOVA including all of the sex steroid manipulations revealed no significant sex differences in CPP responses to ethanol (all *p* > 0.05), however this is not surprising given the sex-specific variability in both CPP after TBI, and the substantial difference in surgical procedures between male and female neonates (T/oil injections in females vs. GDX/Sham surgery in males). To confirm that we have replicated our previously reported findings (Weil et al., 2016b), we conducted an ANOVA to compare CPP responses among control (no sex steroid manipulations) male and female mice, and reveal that control female mice have significantly greater CPP responses to ethanol after TBI compared to intact males (F_1,28_ = 6.022, *p* = 0.022, η_p_^2^ = 0.201). CPP behavior in males and females varied in response to gonadal manipulations and TBI. Among females there was no overall effect of injury (F_1,39_ = 0.335, *p* > 0.05, η_p_^2^ = 0.009, Figure 2A) or of endocrine manipulations (F_2,39_ = 0.574, *p* > 0.05, η_p_^2^ = 0.029). However, there was a significant interaction (F_2,39_ = 4.19, *p* = 0.022, η_p_^2^ = 0.177) such that injury increased CPP responses among control (gonadal unmanipulated) females (F_1,13_ = 5.756, *p* = 0.034, η_p_^2^ = 0.324) but had no effect in any other group (*P* > 0.05 in all cases). Injured females that were treated with T perinatally and then gonadectomized exhibited significantly lower CPP responses than did intact females (F_1,15_ = 6.414, *p* = 0.025, η_p_^2^ = 0.330). In contrast, males exhibited no effects of injury (F_1,36_ = 0.338, *p* > 0.05, η_p_^2^ = 0.009, Figure 2B), endocrine manipulation (F_2,36_ = 1.371, *p* > 0.05, η_p_^2^ = 0.071), or interaction between the two variables (F_2,36_ = 1.976, *p* > 0.05, η_p_^2^ = 0.099).

### Sex Steroid Manipulation Does Not Affect Axonal Degeneration or Microglial Cell Count

**FIGURE 3:**
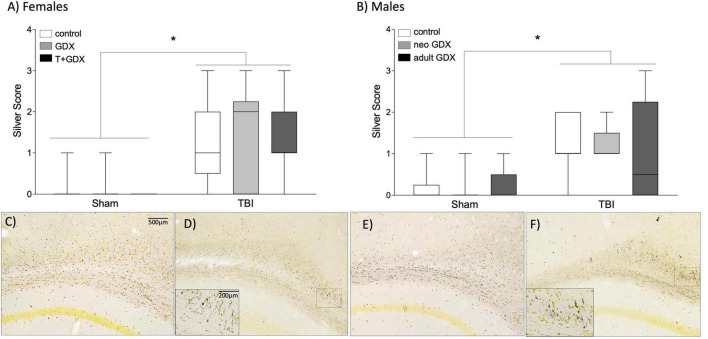
Axon degeneration. Axonal damage scores based on silver staining in **(A)** female and **(B)** male mice. An asterisk (*) indicates statistically significant difference from sham injured mice, ANOVA *p* < 0.05. Boxplots show representative range (minimum and maximum), first quartile, median, and third quartile data for each group. Representative photomicrographs are shown for **(C)** female sham, **(D)** female TBI, **(E)** male sham, and **(F)** male TBI. Scale bar = 500 μm, inset scale bar = 200 μm.

**FIGURE 4:**
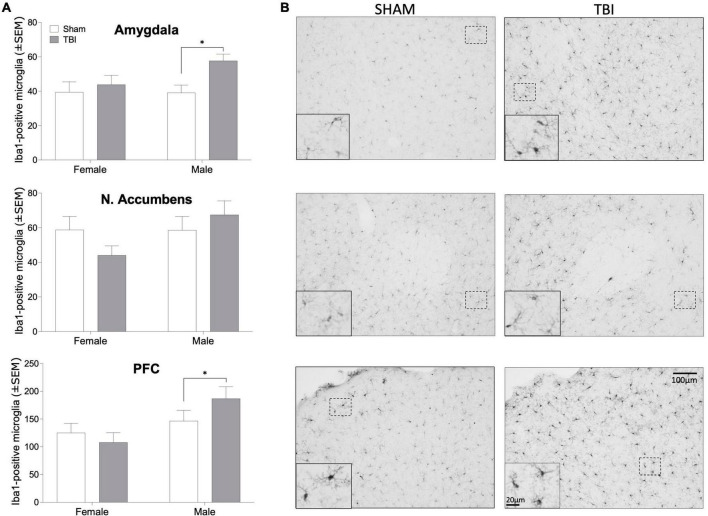
Iba1-positive microglia. **(A)** Bar graphs depicting mean (± SEM) microglial cell counts per mm^2^ in brain regions important for alcohol reward in male and female mice. **(B)** representative photomicrographs of Iba1 immunohistochemistry in male mice. An asterisk (*) indicates significant main effect of injury, ANOVA *p* < 0.05. Scale bar = 100 μm, inset scale bar = 20 μm.

Silver staining was used as indicator of axonal degeneration such that a higher silver score indicated greater axonal degeneration. Injured females (F_1,43_ = 33.16, *p* < 0.0001, η_p_^2^ = 0.435) and males (F_1,35_ = 20.55, *p* < 0.0001, η_p_^2^ = 0.37) exhibited greater evidence of axonal degeneration, as assessed by silver staining than did sham-injured mice (Figure 3). But there was no overall significant sex difference or significant effect of endocrine manipulations among either sex (*p* > 0.05 in all cases) or interactions between the variables. We next examined microglial cell counts in regions of the mesolimbic pathway and prefrontal cortex (PFC), given the known relationship between activity in the amygdala, nucleus accumbens, and PFC and alcohol use disorder (Abernathy et al., 2010; Roberto et al., 2012; Seif et al., 2013; Grodin et al., 2018). Interestingly, microglial cell numbers in key forebrain structures was increased by TBI in injured males but not females (Figure 4). In the amygdala, nucleus accumbens, and PFC of females there were no injury or endocrine manipulation effects, nor were there interactions between the variables (*P* > 0.05 in all cases). In contrast, males, regardless of endocrine manipulation have increased microglial cell counts throughout the forebrain. For instance, in the amygdala (F_1,21_ = 5.404, *p* = 0.35, η_p_^2^ = 0.265) and prefrontal cortex (F_1,21_ = 5.101, *p* = 0.039, η_p_^2^ = 0.254) injured males exhibited significantly greater microglial cell numbers than did sham-injured mice. The nucleus accumbens did not exhibit this effect (F_1,21_ = 1.752, *p* = 0.205, η_p_^2^ = 0.105). A direct comparison of microglial cell numbers in TBI mice between the sexes confirmed a sex difference such that males exhibited significantly greater numbers of microglia in the PFC (F_1, 29_ = 8.036, *p* = 0.009, η_p_^2^ = 0.229), and nucleus accumbens (F_1,35_ = 6.162, *p* = 0.018, η_p_^2^ = 0.157).

## Discussion

Given the noted sex difference in alcohol-related behaviors after TBI in both clinical and basic research, we aimed to gain a deeper understanding of the role of circulating sex steroids as a potential mediator of this phenomenon. Our results represent the first known attempt to delineate the role of sex steroid hormones in drug seeking behavior after TBI. Here we demonstrate that while TBI-induced axonal degradation is not affected by sex steroid manipulations, perinatal testosterone administration and adult gonadectomy in females prevents the development of a conditioned place preference to ethanol. Additionally, although there was an effect of injury on microglial cell count in males, there was no effect of testosterone or gonadectomy at either developmental timepoint on microglia for either males or females. These results represent the first known evidence towards both organizational and activational effects of sex steroids as potential regulators of alcohol-related behavior after TBI in females.

### Masculinization of Females by Sex Steroid Manipulations Eliminates Ethanol Conditioned Place Preference

We hypothesized that sex steroids were the mediators of this sex difference for two primary reasons. First, both neonatal testosterone treatment and adult gonadectomy in female rodents have been shown to affect their response to ethanol. For instance, ovariectomized females drink significantly less than other females (Becker et al., 1985) and estradiol administration increases drinking in ovariectomized mice (Ford et al., 2002). Furthermore, defeminization of female rats through neonatal sex steroid exposure reduces alcohol consumption (Almeida et al., 1998). Secondly, the epidemiological data suggest that women are most at risk of exhibiting later alcohol misuse when a TBI is incurred during puberty (Corrigan et al., 2020).

Sexual differentiation of brain and behavior results from the contribution of appropriately timed steroid hormone exposure (or the lack thereof) and differential gene expression associated with sex chromosome complement (Breedlove and Hampson, 2002). In the current, study we treated female mice with androgens during the early postnatal period. The exposure to androgens, which are aromatized into estrogens in the brain, permanently prevents the development of ovarian cyclicity and other female-typic behavioral patterns. We utilized the CPP paradigm as it specifically assesses the hedonic effects of alcohol that we have previously demonstrated were altered by TBI (Weil et al., 2016b). The current study replicates previous findings: intact female mice injured early in life developed a conditioned place preference to ethanol, while the uninjured females did not (Weil et al., 2016b). Treatment with testosterone neonatally *and* adult gonadectomy abolished the enhanced CPP response to alcohol after TBI. Thus, both female typical sex steroid exposure perinatally and in adulthood are necessary for the TBI-induced potentiation of alcohol reward. In contrast, neither sex steroid manipulations (perinatal or in adulthood), nor TBI was sufficient to enhance alcohol CPP in male mice. Thus, the enhanced CPP response among brain injured females appears to require normal ovarian cyclicity and cannot be replicated by neonatal castration of genetic males.

### Role of Traumatic Brain Injury Pathophysiology as a Regulator of Alcohol-Seeking Behavior

A separate possibility underlying the disparity in alcohol response between males and females is a difference in injury severity. For instance, some data from clinical populations suggest that women fare worse than men after TBI and exhibit greater delays in recovery (Farace and Alves, 2000). However, given that axonal degeneration is not significantly different between unmanipulated males and females after a TBI, we assert that this is not the cause of the sex difference represented in our model. Moreover, axonal degeneration was affected by injury but not significantly altered by neonatal testosterone treatment or gonadectomy. Since the axon injury scores do not mirror the alcohol-related behavior, these behavioral responses appear to occur independently of injury severity.

We also considered that the variations in alcohol response could be due to differential neuroinflammation, as there is an established link between neuroinflammation and substance abuse (Kohno et al., 2019). Moreover, we previously showed that treatment with an inhibitor of microglial activation reduced post-TBI drinking in male mice (Karelina et al., 2018). There are both sex differences in neuroimmune physiology and effects of steroid hormones on immune function (Villa et al., 2016). We had predicted that greater microglial activity in brain regions related to reward processing might correlate with CPP responses among injured mice. Traumatic brain injuries and other early life perturbations can produce a persistent state of increased reactivity to immune challenges (Fenn et al., 2014). Most drugs of abuse, including alcohol can drive inflammatory signaling (Alfonso-Loeches et al., 2010; Lacagnina et al., 2017). There is a substantial literature linking early life stress and adversity with both alterations in microglial physiology *and* increased susceptibility to substance abuse in both preclinical and clinical studies (Enoch, 2011; Crews et al., 2017; Pascual et al., 2017; Johnson and Kaffman, 2018). Taken together, we had reasoned that TBI would prime microglial immune responses such that the administration of alcohol would produce exaggerated inflammatory responses. Sex differences in this relationship could therefore mediate the sex differences in CPP responses to alcohol among injured mice gonadectomy, such that microglial count was greater in the PFC and amygdala of males that underwent a TBI. This is in line with existing evidence that the neuroinflammatory response is greater in males after a TBI (Villapol et al., 2017). One limitation of these experiments is that the length of time between injury and tissue collection (2 + months); thus, testosterone treatment or gonadectomy promote acute changes in neuroinflammation that may resolve over time. Additionally, examining microglial cell numbers only provides a relatively limited amount of information as to the reactivity, gene expression, and secreted factors. This finding suggests that although injury promotes a neuroinflammatory response in males, this occurs independently of sex hormones. Furthermore, this finding reduces the possibility that neuroinflammation is directly responsible for the increase in alcohol consumption after a TBI. neuroinflammatory response in males, this occurs independently of sex hormones. Furthermore, this finding reduces the possibility that neuroinflammation is directly responsible for the increase in alcohol consumption after a TBI.

These data have important implications for case management of individuals with a history of childhood traumatic brain injury. Substance abuse prevention and treatment have the potential to produce significantly better outcomes in this patient population because (1) substance abuse impairs rehabilitation and produces poorer overall outcomes in those with a history of brain injuries and (2) alcohol intoxication is a major risk factor for subsequent TBIs which can have devastating permanent consequences (Weil et al., 2016a,^2019^; Corrigan et al., 2020). Moreover, there is also epidemiological evidence that sex differences exist in the risk of developing substance abuse disorders after brain injury. Humans differ from rodents in that males tend to consume more alcohol whereas the opposite is true among laboratory rodents (Barker et al., 2010). Thus, from a public policy standpoint it will be beneficial to invest in strategies that reduce the risk of developing substance abuse disorders for individuals of both sexes that have experienced TBI. However, these data should highlight the importance of considering the role of sex and sex steroids as potential contributors to substance-abuse related outcomes after brain injuries.

## Conclusion

Overall, these findings provide substantial evidence that sex hormones are involved in the increase in alcohol-related behaviors after a juvenile TBI in female mice. The ultimate reasons for sex differences in the response to alcohol remain unspecified but at a proximate level, ovarian steroids appear to be necessary both perinatally and in adulthood to produce the sex difference in alcohol responses. While this is an important first step to identifying a role for circulating sex steroids as mediators of drug seeking behavior after TBI, there are equally important follow up questions that still need to be addressed to understand the mechanisms by which sex steroids may mediate these behaviors. Key among these is the question of how alcohol use prior to injury affects post-TBI alcohol use, with a particular focus on sex differences. Moreover, future studies are needed to assess the roles of important variables that were controlled in this study, including impact severity and location.

## Data Availability Statement

The raw data supporting the conclusions of this article will be made available by the authors, without undue reservation.

## Ethics Statement

The animal study was reviewed and approved by Ohio State University Institutional Animal Care and Use Committee.

## Author Contributions

ZW and KK contributed to conception and design of the study. JF, RV-C, BW, and RO performed the experiments. RO wrote the first draft of the manuscript. ZW and KK analyzed the data and contributed to the writing and design of the figures. All authors contributed to manuscript revision, read, and approved the submitted version.

## Conflict of Interest

The authors declare that the research was conducted in the absence of any commercial or financial relationships that could be construed as a potential conflict of interest.

## Publisher’s Note

All claims expressed in this article are solely those of the authors and do not necessarily represent those of their affiliated organizations, or those of the publisher, the editors and the reviewers. Any product that may be evaluated in this article, or claim that may be made by its manufacturer, is not guaranteed or endorsed by the publisher.
